# Probiotic Treatment Decreases the Number of CD14-Expressing Cells in Porcine Milk Which Correlates with Several Intestinal Immune Parameters in the Piglets

**DOI:** 10.3389/fimmu.2015.00108

**Published:** 2015-03-10

**Authors:** Lydia Scharek-Tedin, Susanne Kreuzer-Redmer, Sven Olaf Twardziok, Bianca Siepert, Robert Klopfleisch, Karsten Tedin, Jürgen Zentek, Robert Pieper

**Affiliations:** ^1^Institute of Animal Nutrition, Freie Universität Berlin, Berlin, Germany; ^2^Breeding Biology and Molecular Genetics, Humboldt-Universität zu Berlin, Berlin, Germany; ^3^Institute of Molecular Biology and Bioinformatics, Charité-Universitätsmedizin Berlin, Berlin, Germany; ^4^Institute of Microbiology and Epizootics, Freie Universität Berlin, Berlin, Germany; ^5^Institute of Veterinary Pathology, Freie Universität Berlin, Berlin, Germany

**Keywords:** pig, *Enterococcus faecium*, milk, mCD14, intestinal, B cells, T cells

## Abstract

Modulating the mucosal immune system of neonates by probiotic treatment of their mothers is a promising approach which can only be investigated through the use of animal models. Here, we used sows and their piglets to investigate the impact of a bacterial treatment on the sow’s milk and on the neonate piglet intestinal immune system. In previous experiments, feed supplementation of sows with the probiotic *Enterococcus faecium* NCIMB 10415 during pregnancy and lactation had been shown to affect intestinal microbiota and cytokine expression of the offspring during the suckling and weaning periods. We therefore investigated the composition of the milk from treated sows in comparison to samples from a control group. In treated sows, the amount of lactose increased, and the somatic cell numbers were reduced. In all milk samples, the percentage of cells expressing membranous CD14 (mCD14) was greater than the fractions of immune cells, indicating expression of mCD14 on mammary epithelial cells. However, in the milk of *E. faecium*-treated sows, mCD14^+^ cells were reduced. Furthermore, the number of CD14^+^ milk cells was positively correlated with the percentages of B cells and activated T cells in the ileal MLN of the piglets. This study provides evidence for the expression of mCD14 by the porcine mammary epithelium, and suggests an immunological effect of mCD14^+^ milk cells on the piglets’ intestinal immune system. Our study further suggests that mCD14^+^ mammary epithelial cell populations can be modulated by probiotic feed supplementation of the sow.

## Introduction

Probiotic feed supplementation of sows during pregnancy and lactation has been shown to affect the piglets’ intestinal microbiota and the mucosal cytokine levels during the suckling period (1, 2). Early environmental contact and oral uptake of microorganisms excreted with the sow’s feces would be one possible explanation for this observation. However, an altered composition of the sow milk could also be an additional possibility.

Milk has been found to influence the neonate’s development through additional factors other than nutrients, vitamins, minerals, and water. Milk harbors bacteria, antibacterial substances such as lysozyme and lactoferrin, antibodies, cytokines, growth factors, and hormones which are known to be important for the immune defense and the development of the intestinal epithelial cells (IECs) of the offspring [reviewed in Ref. (3)]. Furthermore, milk contains maternal immune cells and epithelial cells. It has been shown that maternal lymphoid cells, delivered through milk, cross the piglets’ intestinal epithelium and are transported to the mesenteric lymph nodes, where they support the piglets’ developing immune system (4). There is evidence to suggest that factors in milk also play a regulatory role in the naïve neonatal immune system. Immunological experience with dietary antigens by the sow is likely to be important for tolerance induction in the offspring, and the ability to distinguish between commensal and pathogenic bacteria appears to be affected by substances delivered through milk (5).

However, in addition to supporting the piglets’ immune system, the immunological components in the milk are also involved in protection of the mammary gland itself. The mucosal surface of the lactiferous gland is vulnerable to microbial infections, and increased numbers of immune cells could also indicate acute infection. In dairy science, determination of somatic cell counts, defined as all cells other than germ or stem cells originating from the cow, has become the gold standard for milk quality. The somatic cell counts recorded for swine milk varies between 2.5 × 10^8^ and 5 × 10^9^ cells/L, which is considered to be relatively high (6–8). In cow milk, cell counts above 2 × 10^8^/L indicate bacterial infections (9). The cellular composition of sow milk has been investigated in several studies (8). Notably, after the first week of lactation, the majority of cells in the sow mammary secretions are reported to be of epithelial origin (8). This is in contrast to milk from healthy cows, where 80% of the somatic cells are immune cells (10, 11). Although epithelial cells are a major component of pig milk, the function of these cells in the piglets is poorly investigated, and the responsiveness of the sow’s mammary epithelial cells to external influences such as probiotic feed supplementation has not been examined.

The epithelial cells of the mammary gland of mice express mCD14 (12), which acts as a co-receptor for the detection of bacterial lipopolysaccharide (LPS) together with the Toll-like receptor 4 (TLR4). Furthermore, the milk of mice, humans, horses, and cattle also harbors soluble CD14 (sCD14) (13–15). Soluble CD14 has been observed to reduce inflammation in the mammary gland and is believed to prevent excessive intestinal inflammation in newborns (16, 17). *In vitro*, sCD14 induces B-cell growth and differentiation independently of activated T cells (18). Fetal IECs express TLR4 and are hyperresponsive to LPS (19). In mice, sCD14 reduces inflammatory responses by blocking circulating LPS thereby limiting the amount of monocyte-bound LPS (20).

With regard to swine, neither the presence of sCD14 in sow milk nor the expression of mCD14 on the porcine mammary epithelial cell has previously been shown. As CD14 expression can be up-regulated by bacterial LPS on macrophages at other mucosal surfaces (21), we were interested to know whether the epithelial cells in porcine milk are mCD14^+^ and whether these cells can be affected by probiotic feed supplementation. To address this issue and to investigate whether the expression of CD14 in the sow milk can be affected by probiotic treatment, we performed a feeding trial with *Enterococcus faecium* and determined the concentration of various nutrients (fat, protein, and lactose), as well as the total somatic cell numbers. Using flow cytometry, the proportion of the total leukocytes, the portion of myeloid immune cells, and the percentage of somatic cells lacking the leukocyte common antigen were analyzed in porcine milk samples. The pan leukocyte marker CD45 was used to distinguish between immune cells and cells of epithelial origin in the sow milk. CD172a (signal regulatory protein alpha) was used to identify myeloid immune cells. Furthermore, the expression of CD14 and CD16 on the milk cells was determined, and we investigated sow milk samples for the presence of sCD14 using Western blot and ELISA.

To determine whether a competitive binding of LPS in the gut of the piglets is a possible function of the mCD14^+^ milk cells, we sought to determine whether other CD14^+^ cells, either IEC or immune cells in the piglets’ intestinal tract, could compete for the binding of LPS. An *in vitro* infection assay was used to investigate whether porcine cells can inhibit an infection of enterocytes with *Salmonella typhimurium*. Furthermore, to investigate the impact of milk on the piglets’ intestinal immune system, we monitored the development of immune cells in several compartments. Due to the fact that the intraepithelial lymphocytes (IELs) are in close contact with both, milk components as well as enterocytes and the intestinal microbiota, we hypothesized that these immune cells could possibly be affected either directly or indirectly in response to changes in bacterial colonization. We therefore monitored the development of different lymphocyte populations in the piglets’ jejunal epithelium, as well as the ileal mesenteric lymph nodes (IL MLN), and the ileal Peyer’s patch (IL PP). Finally, we performed a correlation analysis of the milk data with immune cell parameters and intestinal cytokine expression levels (mRNA) in the piglets.

## Materials and Methods

### Animals

The animal study was approved by the local state office of occupational health and technical safety “Landesamt für Gesundheit und Soziales Berlin” (LaGeSo Reg. Nr. 0347/09).

Twenty four purebred primiparous landrace sows were allocated into either control (*N* = 12) or probiotic groups (*N* = 12) 4 weeks before parturition. The animals were housed under similar conditions but in different stables in order to avoid probiotic cross contamination. Pregnant sows were housed in group pens until 7 days before expected parturition, and then moved to farrowing pens with straw bedding. Farrowing was not induced and cross fostering was performed to balance litter sizes. Piglets were kept with their dams until weaning at the age of 26 ± 2 days. Preparation of IELs and cells from the ileal and mesenteric lymph nodes were obtained from five piglets per group and time point, sacrificed during the suckling period at the age of 12 and 26 days, and after weaning at the age of 35 and 56 days.

An additional 30 piglets were sacrificed at the age of 14, 25, and 35 days to determine the presence of mCD14^+^ cells in the jejunal intestinal tract. These piglets were not part of the feeding experiment and were not fed with *E. faecium*.

### Diets

Diets fed during pregnancy and lactation were formulated to meet the nutrient requirements of pregnant or lactating sows according to GfE (2006). The probiotic *E. faecium* NCIMB 10415 (Cylactin^®^, Cerbios-Pharma SA, Lugano, Switzerland) was mixed into the pregnancy diet at a level of 4.3 × 10^6^ cfu/g and in the lactation diet at a level of 4.2 × 10^6^ cfu/g. Probiotic supplemented feed was offered from day 28 before expected parturition until weaning of piglets on the 26th day of life of piglets. The diets were checked regularly for enterococci by plating on SB medium and by strain-specific PCR for *E. faecium* NCIMB 10415 (22).

### Sampling

Milk was obtained on days 3, 17, and 26 p.p. after stimulation of milk release through i.m. injection with 50 IE of oxytocin (Oxytocin Vet, 10 IE/mL, Veyx-Pharma, Schwarzenborn, Germany). The teats were then washed, and 5 min after the injection, 50 mL milk were obtained by hand-milking.

Six piglets from each group were sacrificed at the ages of 14, 28, and 35 days, and seven piglets per group at the age of 54 days. The number of piglets used for sampling in the different analyses varied from four to seven animals and is indicated in the Section “Results.” For isolation of IEL, a 20-cm section without discrete PP was taken from the mid jejunum. IL MLN were collected as previously described (23). Tissue sections of the JE PP (2 cm) were collected immediately post-mortem and added to RNA later (Ambion) for storage prior to RNA isolations.

### Analysis of nutrients

Twenty milliliters of each milk sample were immediately analyzed for fat, protein, and lactose content by near infrared absorption (Combi-Foss-MilkoScan FT 6000) at the federal milk control laboratories (Landeskontrollverband Brandenburg e.V., Waldsieversdorf, Germany).

### Flow cytometry and fluorescence microscopy

#### Milk cells

Somatic cell counts were determined by flow cytometry in a Combi-Foss-Fossomatic FM FC 500 cytometer (Landeskontrollverband Brandenburg e.V. Waldsieversdorf, Germany). An additional 20 mL of milk was used to isolate cells for determination of cell subpopulations. The samples were first filtered through a nylon mesh (210 μm) and the filtrates were then centrifuged at 340 × *g* for 15 min at 4°C. The cream was skimmed from the top of the tube, and the cell pellets were resuspended in 25 mL of phosphate buffered saline (PBS), and the procedure was repeated. The final cell pellet was then carefully resuspended with a Pasteur pipette in 5 mL PBS, avoiding the fatty deposits, and the cell suspension was then transferred to a second vial. Cells were counted microscopically in a Neubauer chamber, and 10^6^ cells were stained in a two step-protocol using non-conjugated antibodies against CD14, CD45, MHCII, and CD172a (listed in Table 1) followed by secondary antibodies labeled with fluorescein isothiocyanate (FITC) or phycoerythrin (PE). All flow cytometry measurements were carried out with a FACSCalibur™(Becton Dickinson, Heidelberg, Germany) flow cytometry instrument outfitted with a blue laser (488 nm). The BD CellQuest Pro™ Software was used for analysis of the samples.

**Table 1:** **Antibodies used for flow cytometry**.

Specificity	Clone	Isotype	Fluorochrome	Distributor
CD2	MSA4	IgG2a	None	VMRD^a^
CD4α	74-12-4	IgG2b	FITC	Biozol^b^
CD5	9G12	IgG1	None	VMRD^b^
CD8α	76-2-11	IgG2a	PE	Biozol
CD8β	PG164A	IgG2a	None	VMRD
TcR1-N4 (δ)	PGLBL22A	IgG1	None	VMRD
CD14	MIL-2	IgG2b	None	AbD Serotec^c^
CD16	G7	IgG1	None	Acris^d^
CD25	K231.3B2	IgG1	None	Acris
CD45 (allotypic)	MAC323	IgG2a	None	Biozol
(SIRPα, CD172a)	74-22-15	IgG1	None	VMRD
IgM	K521C3	IgG1	None	Biozol
MHCII	MSA3	IgG2a	None	VMRD

*^a^VMRD (Pullman, WA, USA)*.

*^b^Biozol (Eching, Germany)*.

*^c^AbD Serotec (Puchheim, Germany)*.

*^d^Acris (Herford, Germany)*.

For fluorescence microscopy, cells were labeled using antibodies against CD14 and CD45. The secondary antibody against CD45 was labeled with Alexa Fluor 647. Twenty microliters of the cell suspensions (5 × 10^6^ cells/100μL of PBS) were used for microscopy on glass slides under a coverslip. Microscopy was performed with an Olympus BX-41 microscope outfitted with an Olympus U-RFL-T fluorescence unit (Olympus, Berlin, Germany).

#### Intestinal intraepithelial cells

The isolation of the jejunal intraepithelial cells from the tissue samples and the staining procedure were performed as described previously (24). Antibodies used for flow cytometry are listed in Table 1. The following combinations were used for double staining: CD4/CD8β, CD2/CD5, CD5/γδ TcR, and CD8α/γδ TcR. Single staining was carried out for CD16 and CD3. Isolation of immune cells from the IL MLN and cell surface staining of CD4, CD25, and IgM was carried out as previously described (23).

### RNA extraction and real-time PCR

Sample preparations and real-time PCR conditions have been previously described (2). Briefly, tissue samples of ileal and jejunal Peyer’s Patches (20 mg) were homogenized in buffer provided in the RNeasy Mini Kit (Qiagen) and RNA extracted from the resulting tissue homogenates with the RNeasy Mini Kit using RNase-free DNase according to the manufacturer’s recommendations (Qiagen, Hilden, Germany). Two micrograms of purified total RNA was used for reverse-transcription into cDNA with MMLV reverse-transcriptase and random hexamer oligonucleotide primers according to the manufacturer’s instructions (Promega, Mannheim, Germany). Gene-specific primers included porcine beta-actin (β-ActinF, GGACTTCGAGCAGGAGATGG; β-actinR, GCACCGTGTTGGCGTAGAGG) (25) and porcine IL-8 (poIL8F-2, TTCGATGCCAGTGCATAAAT; and poIL8R-2, CTGTACAACCTTCTGCACCCA) (26).

Real-time PCRs were performed using a StepOnePlus™ Real-time PCR System and Power SYBR^®^ Green PCR Master Mix (Applied Biosystems, Darmstadt, Germany). Three-step amplifications were performed using 2 μl of cDNA (dilution: 1:10) template in reactions consisting of denaturation at 95°C for 10 min, followed by 40 cycles of denaturation (95°C, 15 s), annealing (gene-specific, 55–58°C, 30 s), and elongation (72°C, 30 s). β-Actin (ACTB) was chosen as the reference gene based on geNorm, BestKeeper, NormFinder software analyses of qRT-PCR (27, 28) as well as determinations comparing ACTB and GAPDH (data not shown). Efficiencies of the RT-PCR reactions ranged from 90 to 95%. Negative controls included mock reactions without reverse-transcriptase and PCR reactions using purified total RNA to exclude genomic DNA contaminations.

The relative changes in gene expression was determined using the 2^−ΔΔ^*^C^*^t^ method (29) where the average cycle threshold (*C*_t_) values of replicates were calculated, and the *C*_t_ values relative to ACTB controls (Δ*C*_t_) were computed for each gene. ΔΔ*C*_t_ was derived by subtracting the average Δ*C*_t_ for the control group and fold differences were then determined as 2^−ΔΔ^*^C^*^t^ for each gene.

### ELISA

The milk samples (3 mL) were centrifuged at 300 × *g* at 4°C for 10 min. The cell-free interphase beneath the fatty upper layer was removed using a needle and stored at 4°C for later processing. The cell pellet was resuspended in 1 mL of PBS, transferred into a clean vial, and centrifuged again. The washing procedure was repeated twice discarding the supernatant. The final, washed cell pellets were resuspended in 500 μL of PBS. Two-hundred fifty microliters of this suspension were treated with 1 μL of Triton X-100 (Sigma-Aldrich, Steinheim, Germany) and 10 μL PBS, the remaining 250 μL was treated with Triton X-100 and 10 μL of 10% porcine bile extract (Sigma-Aldrich).

The milk supernatant was divided into two aliquots of 500 μL each. One aliquot received 2 μL of Triton X-100, the second sample received 20 μL of 10% porcine bile extract. All four samples (two skimmed supernatant and two cells suspensions) were shaken for 30 min at RT. To detect CD14 in milk samples, a sandwich ELISA was used (MBS739747, Emelca, Breda, Netherlands) following the manufacturer’s instructions with an additional washing step included after incubation of the samples on the coated plates.

### Blocking the staining of mCD14 on blood monocytes using milk supernatant

The same monoclonal antibody used to stain the milk cells (MIL-2) was also used for the monocyte staining. Before addition of the antibody to the monocytes (10^6^ cells in 30 μL of PBS), the antibody (0.5 μL) was incubated either in 70 μl of either pure milk supernatant, or 50 or 10% dilutions of milk supernatant in PBS.

### *In vitro* infection assay with *Salmonella*

To determine the effects of milk cells on *Salmonella* infection of IECs, the porcine jejunal epithelial cell line IPEC-2J was infected with *S. typhimurium* DT104 (BB440) harboring a green fluorescent protein (GFP)-expressing plasmid derived from strain SMØ22 (30). The cell suspension (10^6^ IPEC-J2 cells in 1 mL RPMI medium) were infected with 10^7^ bacteria which had been pre-incubated for 30 min at 37°C in 1 mL of RPMI or in 1 mL RPMI containing 10^6^ milk cells. After pre-incubation, the bacteria:milk cell mixtures were added directly to the IPEC-J2 cells.

### Statistical evaluation

To determine possible correlations between the milk data and immunological data measured in samples from the piglets, statistical Pearson correlation coefficients were calculated with R version 3.1.0. Furthermore, the relationship between relative IL-8 expression and a basal CD14^+^ value for each sow was calculated by a linear regression model. The CD14^+^ ratio was corrected for measurement time points and probiotic treatment by the application of a linear mixed model.

## Results

### Milk nutrients

At day 26 of lactation, milk samples of sows fed with *E. faecium* showed higher concentrations of lactose (Table 2). The concentrations of fat and protein did not differ between the groups.

**Table 2:** **Milk composition (gram per liter) of control and *Enterococcus faecium* NCIMB 10415 feed supplemented sows during the suckling period**.

	Control	EF	*P*-value
**Day 3 post partum**
Fat	113 ± 17	109 ± 30	0.682
Protein	65 ± 12	58 ± 5	0.070
Lactose	44 ± 5	49 ± 5	0.058
**Day 17 post partum**
Fat	95 ± 13	89 ± 17	0.351
Protein	50 ± 4	53 ± 6	0.183
Lactose	53 ± 2	53 ± 3	0.479
**Day 26 post partum**
Fat	82 ± 25	95 ± 21	0.155
Protein	53 ± 5	53 ± 5	0.923
Lactose	47 ± 7	52 ± 2	**0.030**

### Cellular composition of milk

**Figure 1:**
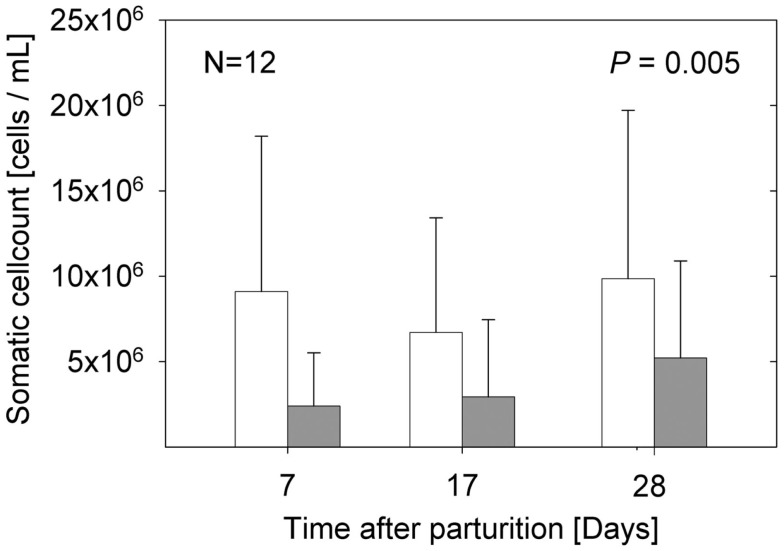
**Total somatic cell counts of milk samples from sows in the control group (open bars) and sows fed with *E. faecium* (filled bars)**. Bars illustrate arithmetic means with respective SD. The group difference over all time points is expressed by the significance level (*P* = 0.005).

**Figure 2:**
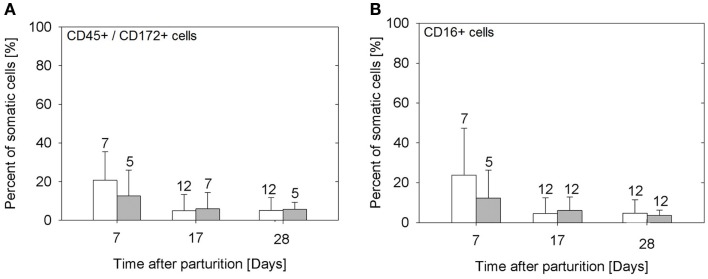
**Percentage of myeloid (CD45^+^/CD172a^+^) cells (A), and of cells expressing the FcγR III (B) in the milk of sows in the control group (open bars) and in the probiotic group (filled bars)**. Bars illustrate arithmetic means with respective SD. The number of samples analyzed per group is indicated with the numeral upon the error bar. Values of CD45^+^/CD172a^+^ cells decrease from day 7 to later time points (*P* = 0.0019), as do the percentages of cells expressing FcγR III (*P* = 0.001).

The samples from the sows fed *E. faecium* showed significantly lower cell numbers compared to the samples from the control sows (*P* = 0.005). The percentage of lymphocytes (CD45^+^/CD172a^−^) was small, below 3 in 94% of the milk samples (data not shown). While the total numbers of somatic cells were not significantly different between the three time points examined (Figure 1), the portion of myeloid immune cells (CD45^+^/CD172a^+^) decreased significantly from day 7 to day 17 (*P* = 0.0019; Figure 2A) without apparent group differences. A substantial part of the immune cells expressed the Fcγ receptor III (CD16; Figure 2B).

**Figure 3:**
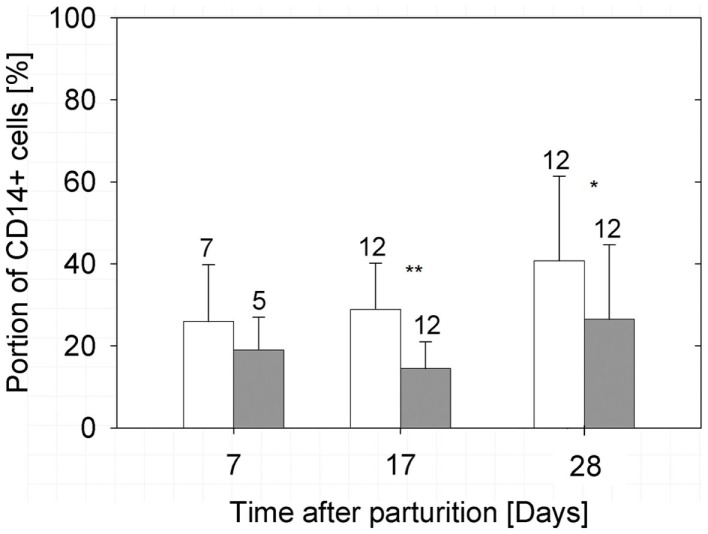
**Percentages of CD14-positive cells in the milk of treated (filled bars) and non-treated (open bars) sows**. The bars illustrate arithmetic means with respective SD. The number of samples analyzed per group is indicated by the numeral at the error bar. A strong significant difference (*P* < 0.01) and a highly significant difference (*P* < 0.001) between the groups are indicated by one or two asterisks, respectively.

While the fraction of CD45^+^ immune cells decreased during lactation, the percentage of cells expressing membranous CD14 (mCD14) did not, and the number of mCD14-expressing cells clearly exceeded the number of immune cells in the milk samples (Figure 3). Comparing the samples from both groups, the sows fed *E. faecium* showed a lower percentages of mCD14^+^ cells in the milk (*P* = 0.01 over all measurement time points and *P* < 0.001 at day 17 of lactation). Based on morphological criteria, the CD45^+^/CD172a^+^ myeloid cells are relatively small with high internal complexity. Cells positive for CD16 belonged to this immune cell population (Plots 2–5 in Supplementary Material). A large fraction of these cells also expressed CD14^+^. However, the expression of CD14 was also recognizable in cells lacking the leukocyte common antigen CD45. These cells were clearly larger. (Plots 6–13 in Supplementary Material).

Despite the reduction of CD45^+^ immune cells in the milk samples to average values below 6% at day 17 of lactation (Figures 2A,B), a high percentage of cells, remained positive for CD14 in the milk samples. In samples of the control group in average, 41% of the cells were CD14^+^/MHCII^−^ at the end of the lactation period (Figure 3; Table S1 in Supplementary Material).

### Fluorescence microscopy of milk cells

**Figure 4:**
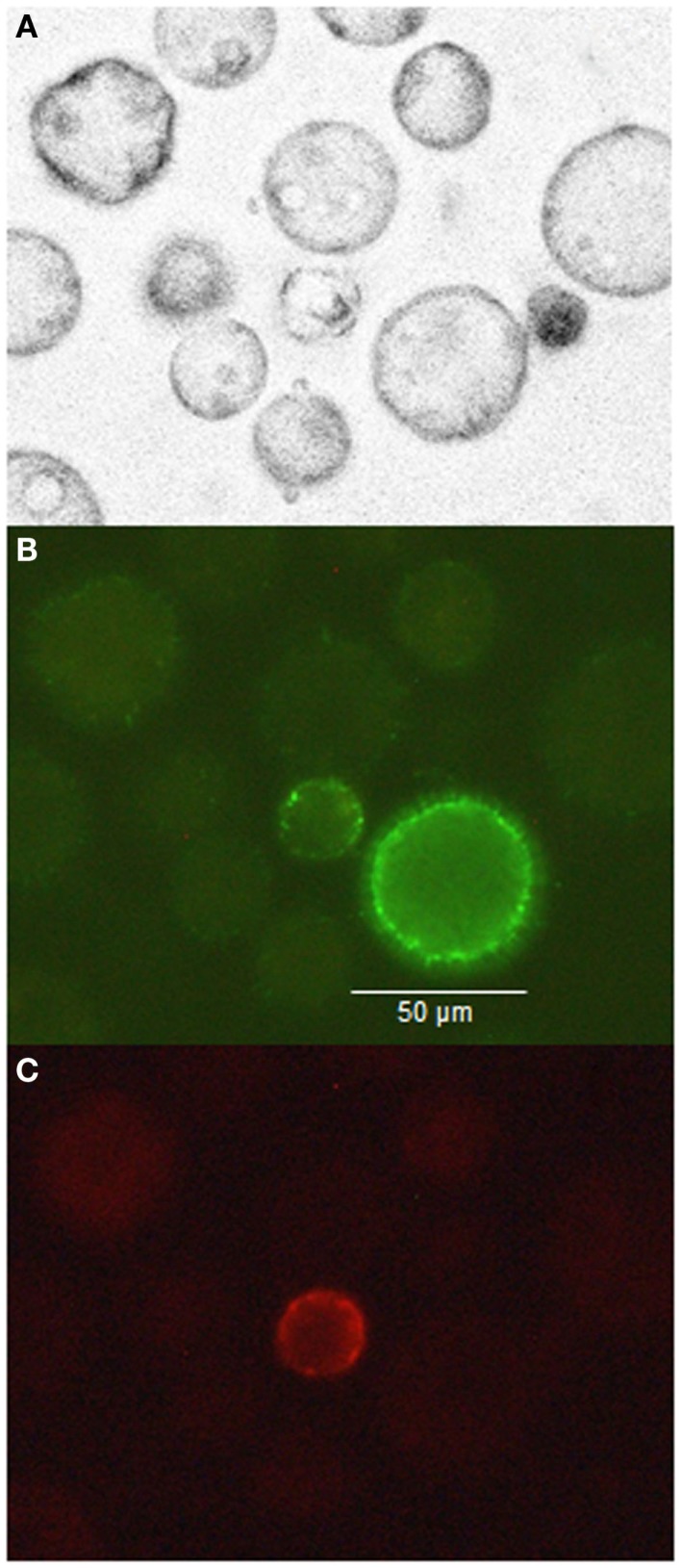
**Milk cells marked with antibodies (anti-CD14 with FITC and anti CD45 with PE) visualized natively (A), using a 530-nm band pass filter for FITC (B), and using a 585-nm band pass filter for PE (C)**.

In samples of milk cells, the large morphology, CD14^+^ cell population showed no expression of CD45 (PE, Figure 4C), and a somewhat variable expression of CD14 (FITC) at the cell surface (Figure 4B). Leukocytes expressing CD45^+^ were clearly smaller (Figure 4C).

### Immune cells in the piglets’ GALT

**Figure 5:**
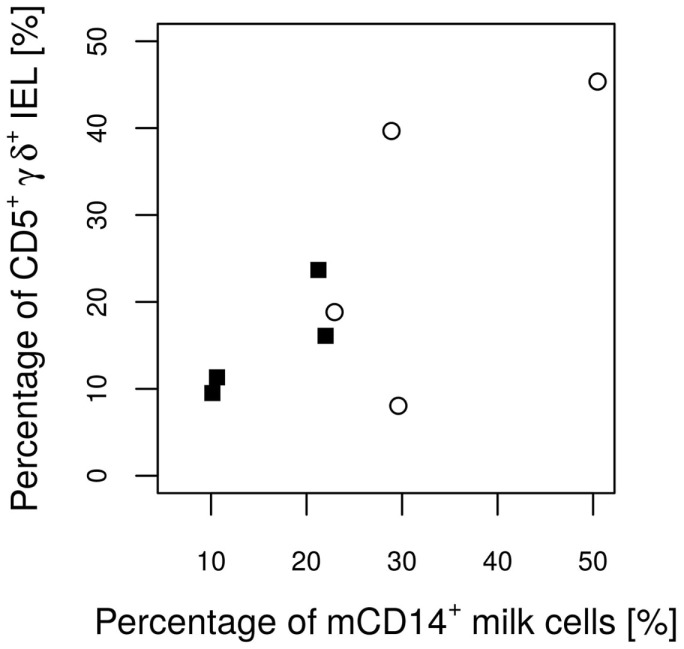
**Percentages of CD5^+^ γδ T cells from all IEL in the piglets’ epithelium on day 26 in relation to the CD14 value of the attendant sow milk measured on day 17 of lactation**. The Pearson correlation coefficient (ρ = 0.77) indicates a significant (*P* = 0.027) linear positive dependence.

**Figure 6:**
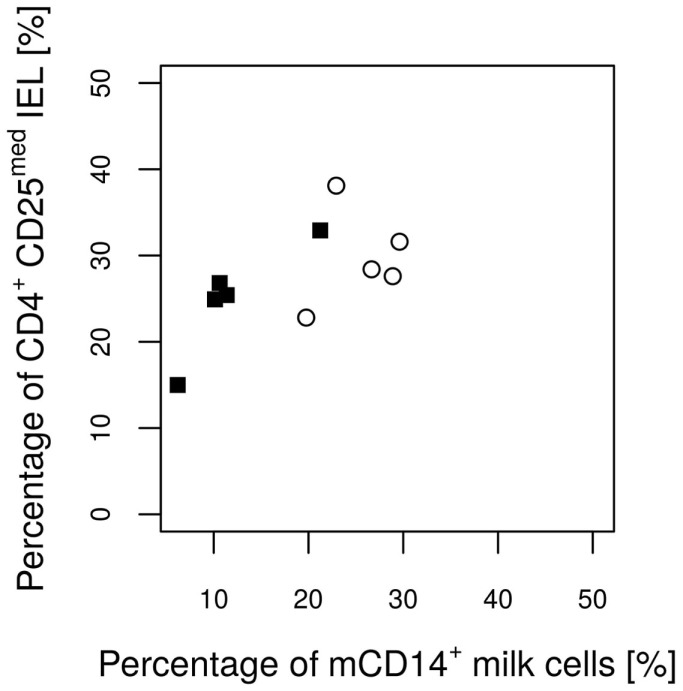
**Percentages of CD4^+^ CD25med T cells from total lymphocytes in the IL MLN of the piglets on day 12 in relation to the CD14 value of the attendant sow milk measured on day 17 of lactation**. Values of the control group and the *E. faecium* group are pictured with filled squares or blank circles, respectively. The Pearson correlation coefficient (ρ = 0.64) indicates a significant (*P* = 0.047) linear positive dependence.

**Figure 7:**
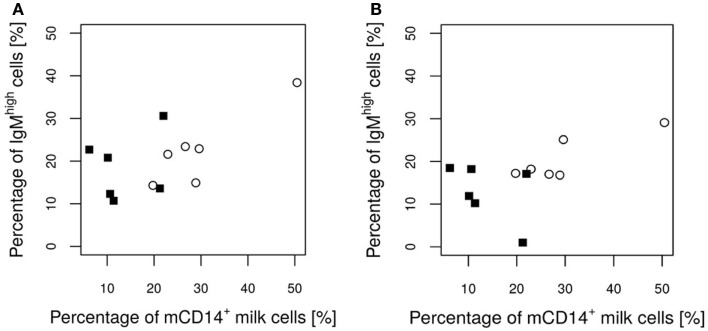
**Percentage of IgM^+^ cells in the IL MLN of the piglets after weaning in relation to the CD14 value of the attendant sow milk measured on day 17 of lactation**. Values of the control group and the *E. faecium* group are pictured with filled squares or blank circles, respectively. At day 34 **(A)**, a significant positive correlation (*P* = 0.025) exists. On day 54 **(B)**, the *P*-value indicates a tendency (*P* = 0.065) for a positive correlation.

**Figure 8:**
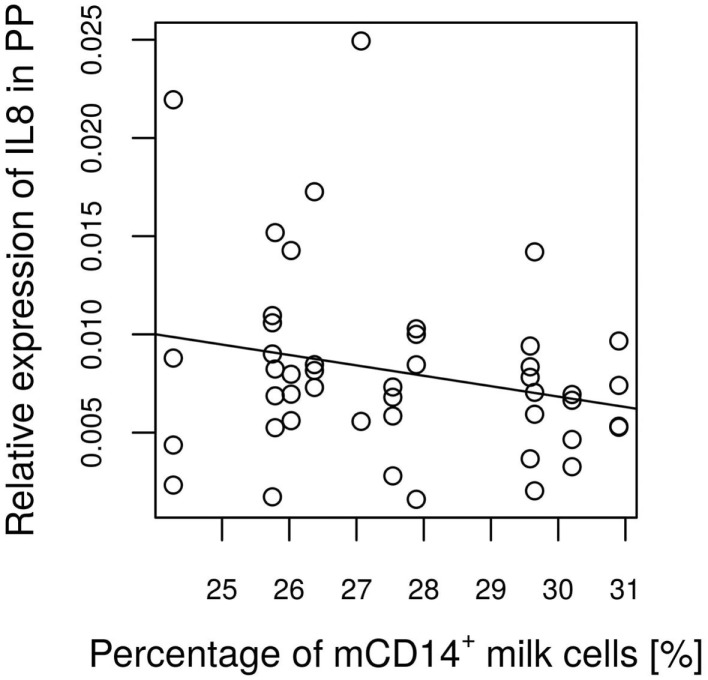
**Expression of IL-8 in relation to a basal CD14 value of the attendant sow milk at every time point measured**. The linear regression coefficient between IL-8 expression and the basal CD14 value indicate a tendency (*P* = 0.136) for a negative relationship.

No significant differences were found between the groups with regard to the various IEL populations (Plots 20–24 in Supplementary Material). However, based on statistical correlations, a linkage between the number of CD14^+^ milk cells and the development of the piglets’ intraepithelial γδ T cells appears probable. As the most significant group differences regarding the concentration of CD14-expressing milk cells were observed on day 17 of lactation, this time point was chosen to investigate possible correlations with immune parameters in the piglets. Shortly before weaning, at the age of 26 days, a positive correlation between CD14^+^ milk cells and the percentages of CD5^+^ γδ T cells in the piglets’ jejunal epithelium was observed (Pearson correlation ρ = 0.77, *P* = 0.027; Figure 5). For the same time point, similar results were found for CD8^+^ γδ T cells (ρ = 0.59, *P* = 0.127, data not shown). Furthermore, in 12-day-old suckling piglets, the cell populations in the ileal mesenteric lymph nodes (IL MLN) may have been affected by the milk composition such that the percentages of CD4^+^ CD25med cells were higher in those piglets that had access to milk with higher numbers of CD14^+^ cells (ρ = 0.64, *P* = 0.047; Figure 6). After weaning, piglets from sows with high CD14 values in the milk showed significantly higher percentages of cells expressing IgM in the IL MLN (ρ = 0.64, *P* = 0.025; Figure 7A and ρ = 0.55, *P* = 0.0062; Figure 7B). While a significant negative relationship between CD14^+^ milk cells and the expression of IL-8 was not verified statistically, an apparent negative correlation was nevertheless noticeable in the jejunal PP (ρ = −0.48; *P* = 0.136; Figure 8).

### Expression of mCD14 on the intestinal epithelium of piglets

In *ex vivo* cell isolations of IECs from suckling and weaned piglets, CD14^+^ cells were clearly detectable (Plots 14–19 in Supplementary Material). All CD14^+^ cells were additionally positive for the leukocyte common antigen CD45. Mean values of intraepithelial CD14^+^ cells (arithmetic mean and SD) for piglets aged 14, 24, and 35 days were 29.4% (±29.1), 16.1% (±12.8), and 14.4% (±4.2), respectively.

### ELISA

In skimmed and cell-free supernatants from porcine milk, no CD14 was detected via ELISA, whereas cell suspensions treated with Triton X-100 showed positive responses. However, the levels determined did not correlate with the dilutions. Although OD values plateaued at a value of 0.2, which was within the linear range of the standard curves, the values did not follow step-wise decreases between dilutions of 1:200, 1:400, and 1:800, but rather remained at a constant plateau value. The addition of bile extract from swine did not lead to significantly higher detection levels.

### Blocking the cellular staining of mCD14 using milk supernatant

Staining of whole milk with anti-CD14 was not possible. Cells were not clearly positive. Furthermore, the addition of increasing concentrations of cell-free milk supernatant to porcine PBMC inhibited the staining of mCD14 on the monocytes in a concentration-dependent matter.

### *In vitro* infection assay

After *in vitro* infection of intestinal epithelial IPEC-J2 cells with GFP-expressing *Salmonella*, a distinct portion of the IPEC-J2 cells (36.2%) acquired fluorescence properties in FL1, indicating infection, whereas the remaining cell population remained uninfected. Pre-incubation of the GFP-expressing bacteria with milk cells prior to the *in vitro* infection of the IPEC-J2 cell culture led to a reduced infection rate of the IPECs (13.3%). Concomitant with the reduced infection rate of the IPEC-J2 cells, an increase in fluorescent cells were found in the milk cell gate (>55%). Whether the GFP^+^ *Salmonella* infected the milk cells, i.e., were intracellular, or were attached to the milk cells was not determined.

## Discussion

Analysis of the cell composition in the sow milk revealed unexpectedly high numbers of mCD14-expressing cells. The proportion of macrophages in cell suspensions of sow milk has been reported to range from 5 to 9% (8, 31). However, in our milk samples of the control group, an average of 41% of somatic cells was found to be positive for mCD14. Simultaneous analysis of CD14 and CD45 revealed that most of the mCD14^+^ cells were negative for CD45 and thus were neither myeloid nor lymphatic cells. As mentioned previously, the majority of the cells in the sow mammary secretions are of epithelial origin after the first week of lactation (8). As CD14-expressing epithelial cells have been described for the mammary gland of mice (12), we assumed that the majority of mCD14^+^ cells in the porcine milk were also of epithelial origin. These large, mononuclear CD14^+^ cells were morphologically clearly distinguishable from small, mononuclear, CD45^+^ lymphocytic cells or small, polymorphonuclear granulocytes (Figures 4A–C; Plots 2–7 in Supplementary Material). At the beginning of lactation period (day 7), a considerable amount of immune cells was detected in the sow milk (above 30% in three animals). Unfortunately, the portion of the immune cells expressing CD14 was not determined at this time point. However, from the 17th day of lactation, the majority of CD14^+^ cells in the milk are of epithelial origin, since the percentage of immune cells decreased to an average of less than 6%. In a dual staining, the existence of the CD45^−^/CD14^+^ cells was verified via both flow cytometry and fluorescence microscopy (Figure 4; Plots 8–13 in Supplementary Material).

With regard to the possible function of epithelial mCD14, two possibilities appear plausible. The attachment of bacteria to cells released into the milk might represent a cleaning function in the lactiferous glands, i.e., a means of removing bacteria to prevent colonization. However, these cells could also have another, additional function in the intestinal tract of the offspring. We initially suggested that these cells could possibly inhibit the attachment of bacteria to the LPS receptor expressed on IECs of the new borne piglet and thereby reduce the risk of intestinal inflammation during the first weeks in life. In the porcine intestinal epithelial IPEC-J2 cell line, the expression and the regulation of TLR4 after infection with an enteric virus has been reported (32). In 35-day-old weaned piglets, a very low level of expression in the jejunal tissue has been reported in comparison to the lung and the spleen (33). We determined the occurrence of CD14 on cells isolated from the intestinal epithelium of suckling and weaned piglets and found only CD45^+^ immune cells expressing mCD14. However, based on our determinations, we cannot exclude the expression of mCD14 on IEC. This may depend on the translocation of bacteria through the epithelial barrier and/or contact with the basolateral side of the IEC. It may also be the case that mCD14 is not expressed at the surface of IEC but that soluble CD14 (sCD14) is secreted by enterocytes. Independent of the presence of CD14 in IEC, we detected a considerable fraction of intraepithelial immune cells expressing mCD14 in the piglets. From morphological criteria, these cells were probably neutrophils (Plot 24 in Supplementary Material). Thus, regardless of variable levels of expression of mCD14 on enterocytes, a competitive function for the mCD14^+^ cells from sow milk for intestinal LPS may be possible, particularly during gut inflammation. It should be mentioned that the values for CD14^+^ epithelial immune cells in piglets aged 14 days showed large variations (7.8–77.2%), and the very high numbers of CD14^+^ immune cells in two of the young piglets were likely symptoms of a gut inflammation. The number of investigated piglets per time point (*N* = 7) was too low to draw conclusions about the physiological levels of CD14^+^ cells in the intestinal epithelium of piglets.

Our results suggest that the CD14^+^ milk cells may compete with the piglets’ immune cells for the binding of LPS. An expression of CD14 by enterocytes was not visible. To test the hypothesis of a possible competitive binding of bacterial LPS by milk cells, we performed *in vitro* assays and found that *Salmonella* indeed binds to milk cells and that the binding interferes with the *in vitro* infection of enterocytes IPEC-J2 cells. Based on these observations, we suggest an impact of CD14^+^ milk cells on the piglet’s bacterial colonization appears possible. It has been reported that feeding of *E. faecium* to sows led to changes in the bacterial colonization of the sows’ as well as of the piglets’ intestinal tract (34). A carry-over of bacterial populations from the sow to the offspring has been considered to be likely. However, the observed changes of bacterial populations in the piglets did not mirror the quantitative composition of the mothers’ microbiota. Our new results might suggest that the CD14^+^ cells in the milk may affect the bacterial communities in the offspring. The correlations between CD14^+^ cells in the milk and the immunological parameters in the piglets might have been indirect effects mediated by a shifted micriobiota.

Whether membrane-bound CD14 *in vivo* reaches the intestinal lumen in an intact form is unknown. Experiments with human feces showed that sCD14 was absent from the stools of breast-fed infants. *In vitro* digestion analyses suggested that sCD14 is likely to survive the pepsin digestion but is probably destroyed by pancreatin (17). Based on these findings, the authors concluded that the presence of intact sCD14 in breast milk could promote innate immunity only in the low bacterial density lumen of the upper digestive system. To date, the existence of mCD14 in the milk of pigs has not been described, and the stability of the protein in the digestive tract of suckling piglets has not been investigated. It therefore remains unclear whether the *in vitro* results reported here reflect a mechanism that is relevant for *in vivo* conditions.

The possible functions of sCD14 remain controversial. It has been reported to have a neutralizing, anti-inflammatory role and protect against LPS-induced cell death (35, 36). In our study, an apparent negative correlation between CD14^+^ cells and IL-8 expression in the jejunal PP of the piglets was not supported statistically. The highly interesting possibility of an anti-inflammatory effect therefore remains an open question. Whether CD14 is shed from cells in the sow milk before or after the uptake of the milk by the suckling piglets, is not known. Tangible evidence for the presence of sCD14 in swine milk is missing in prior studies. Using Western blotting, we were unable to detect a protein band reacting with anti-CD14. However, this observation does not provide evidence for the absence of sCD14 in the liquid phase of milk; the epitope may be destroyed under denaturing conditions. However, results from ELISA assays led us to conclude that sCD14 is likely not present in porcine milk. Based on the observation that bile extract did not improve the detection, it appears improbable that CD14 is retained within micelles. However, it may not be solved in the buffer used, but rather remains bound to the cell membranes.

The fat globules in milk are initially surrounded by cell membrane which is released from the apical side of the mammary cell (37). When mammary cells are destroyed or membrane-bound globules break down, the CD14^+^ membrane fragments can be shed into the milk. This we consider to be a likely explanation as to why it has not been possible to stain CD14^+^ mammary epithelial cells in whole milk. The membrane-bound globules and membrane fragments may block the anti-CD14 antibody, and the CD14^+^ cells in the milk are accompanied by an unknown amount of CD14^+^ membrane fragments. These membrane fragments should be the subject of future investigations.

Previous studies with *E. faecium* fed to sows and piglets did not indicate an impact of the probiotic treatments on the histological structure of the jejunum, in terms of changes in the villus length or depth of the crypts. However, an impact on the epithelial immune cells, immune-associated gene expression patterns, as well as on the bacterial colonization has been reported (2, 24, 34, 38). In the present feeding study, we observed a positive correlation between CD14^+^ cells in the milk and the frequencies of CD5^+^ γδ T cells, determined as a percentage of all intraepithelial immune cells (Figure 5). Knowledge about the function of porcine intraepithelial γδ T cells remains limited. Unlike circulating γδ T cells, they express the natural killer receptor, NKG2D, and are therefore suited for recognition of stressed epithelial cells in the gut (39, 40). In experiments with mice, it has been reported that the absolute numbers of intraepithelial γδ T cells are not affected by microbial colonization (41). As we determined the relative amounts within total IEL, it remains possible that CD14^+^ in fact hampers the increase of IEL other than γδ T cells. However, we were unable to identify a significant negative correlation to any other IEL population in our study. Another immunological parameter in piglets that was positively correlated with CD14^+^ milk cells were CD4^+^/CD25med cells in the ileal MLN. These cells are reported to be activated T cells (42). This observation may be linked to significantly higher values of IgM^+^ cells in the IL MLN of the same animal group at a later time point.

From *in vitro* studies, it is known that sCD14 in colostrum and milk is a B-cell mitogen. Filipp and co-workers have suggested that it may be important for the activation of B cells before full functional helper T cells are generated (18). The mitogenic impact of CD14 on B cells *in vivo* proposed by those authors may be supported by our animal study. The numbers of CD14^+^ milk cells were positively correlated with the percentages of activated T cells during the early suckling phase and were also positively correlated with the numbers of IgM-expressing B cells in the IL MLN after weaning. In a pediatric study, a similar correlation between sCD14 in the colostrum and the numbers of circulating immunoglobulin-secreting cells in 12-month-old children was found (43). As piglets are born with a relatively immature adaptive immune system (44), this function may be particularly important for this species during the first weeks in life. Furthermore, CD14^+^ milk cells appear to prevent the *in vitro* infection of the intestinal epithelium with *S. typhimurium*. The stability of mCD14 transferred into the intestinal tract of the piglet on the surface of fat-producing cells is an issue that should be addressed in further *in vitro* and *in vivo* investigations.

From our work, we conclude that high numbers of epithelial cells expressing mCD14 are indeed present in sow milk, and feeding a probiotic strain of *E. faecium* to sows changes the composition of the sow milk. We observed reduced cell numbers and lower percentages of CD14^+^ cells in the supplemented animal group. Furthermore, the reported early influence of *E. faecium* on the piglets’ immunological development appears, at least to a certain extent, to be transferred by the milk. In our study, lower numbers of CD14^+^ cells in the milk of sows fed with *E. faecium* were correlated with lower numbers of activated T cells and IgM^+^ B cells in the IL MLN of the offspring. However, it remains possible that the observed effects and correlations may have been indirect, mediated by a changed bacterial colonization which was less challenging for the immune system of the piglets. While our current study does not allow discrimination between these possibilities, future studies may contribute to a better understanding of the role of probiotics, maternal CD14, and piglet health.

## Author Contributions

RP and LS designed the animal experiment and collected the samples from sows and piglets. The flow cytometry analysis was carried out by SK and LS. BS and KT performed the RNA extractions and the quantitative RT-PCR. ST performed the statistical analyses and contributed to the interpretation of the data. JZ conceived the project and interpreted the results. RK provided specialized expertise in microscopy of milk cells. The manuscript was written by LS with the contributions of all other authors.

## Conflict of Interest Statement

The authors declare that the research was conducted in the absence of any commercial or financial relationships that could be construed as a potential conflict of interest.

## Supplementary Material

The Supplementary Material for this article can be found online at http://www.frontiersin.org/Journal/10.3389/fimmu.2015.00108/abstract
