# Supplementary material for: Probiotic Treatment Decreases the Number of CD14-Expressing Cells in Porcine Milk Which Correlates with Several Intestinal Immune Parameters in the Piglets
Source: Front Immunol. 2015 Mar 10;6:108. doi: 10.3389/fimmu.2015.00108 (PMC4354412; doi:10.3389/fimmu.2015.00108)
Supplement: Supplementary file 1 [file data_sheet_1.zip › Supplementary_Plots.pdf]

Milk sample

(day 7 of lactation, *E. faecium* group)

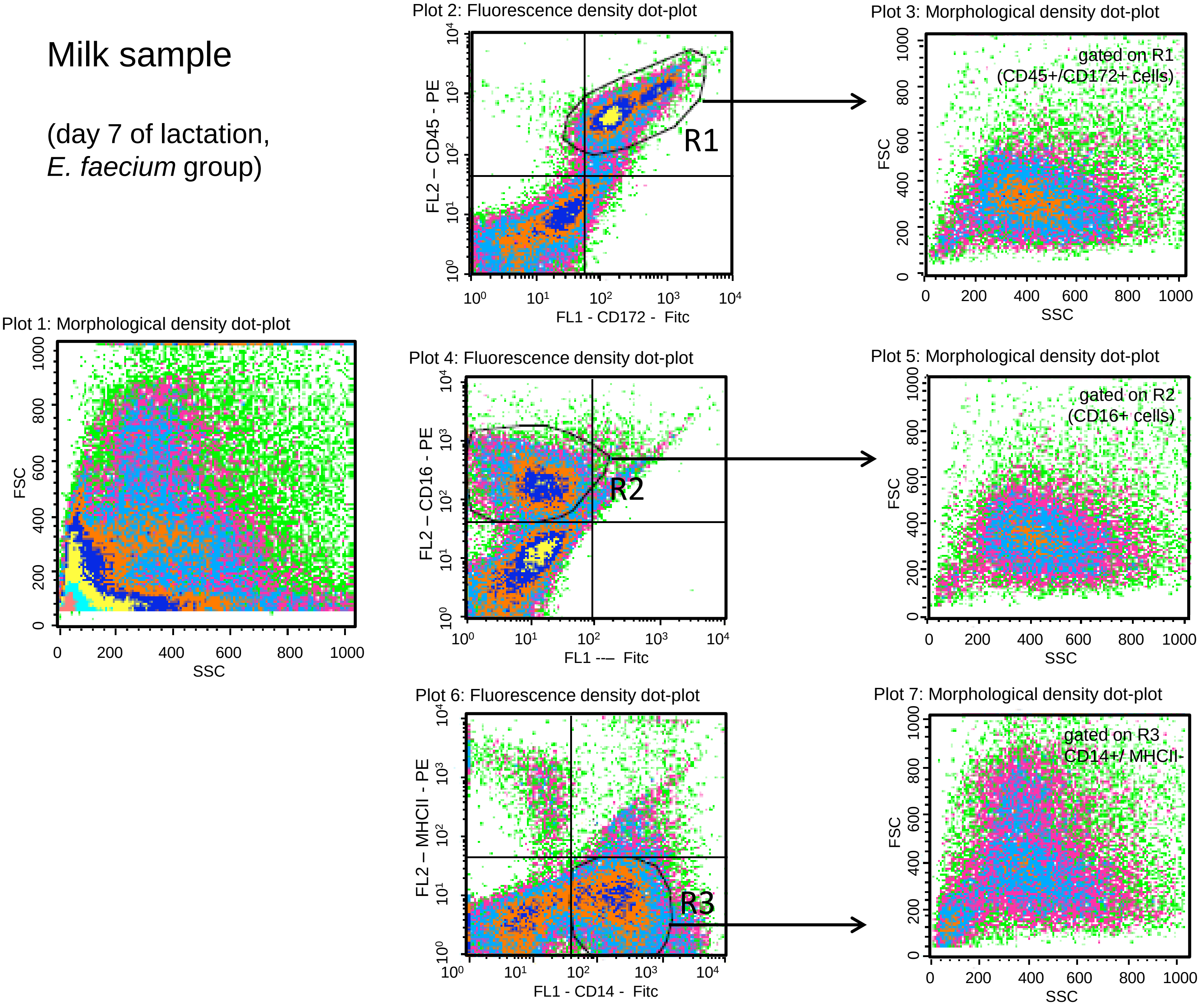

Milk sample

(day 6 of lactation,
no feeding group)

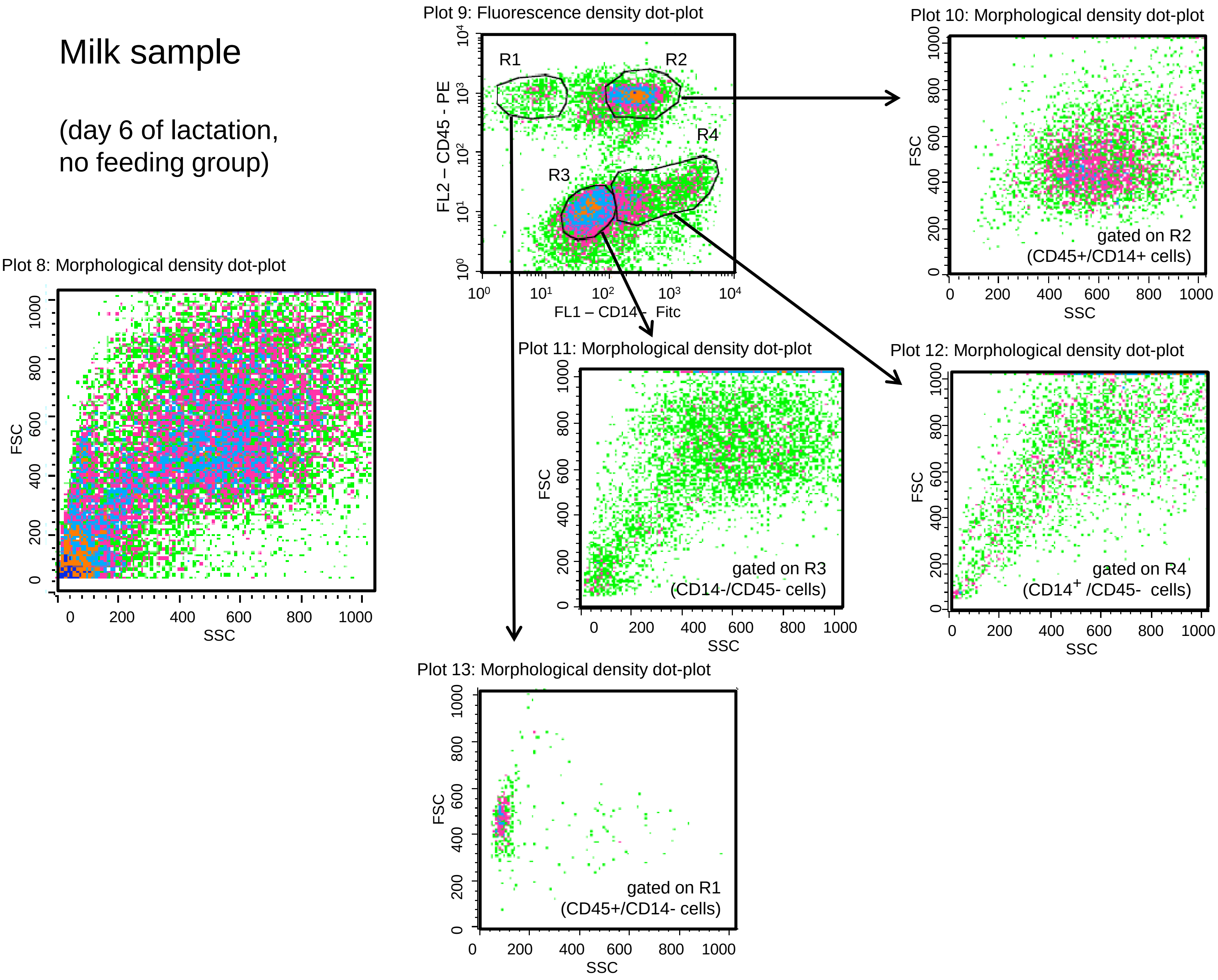

Jejunal intraepithelial cells

(piglet aged 28 days,
control group)

Plot 15: Fluorescence density dot-plot (gate L)

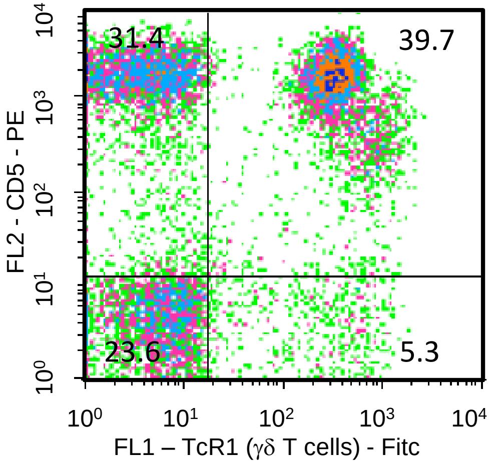

Plot 16: Fluorescence density dot-plot (gate L)

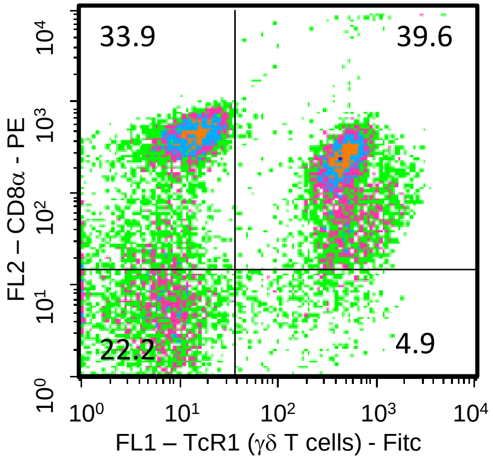

Plot 14: Morphological density
dot-plot with leukocyte gate (L)

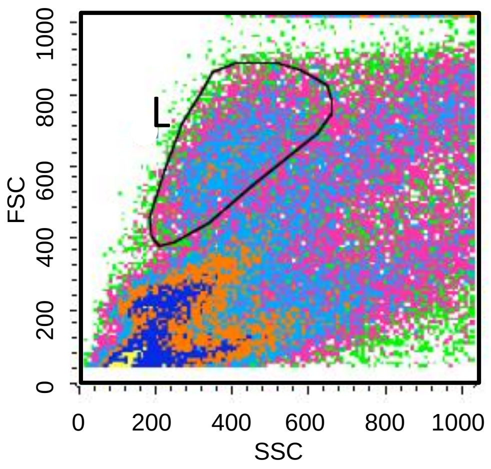

Plot 17: Fluorescence density dot-plot (gate L)

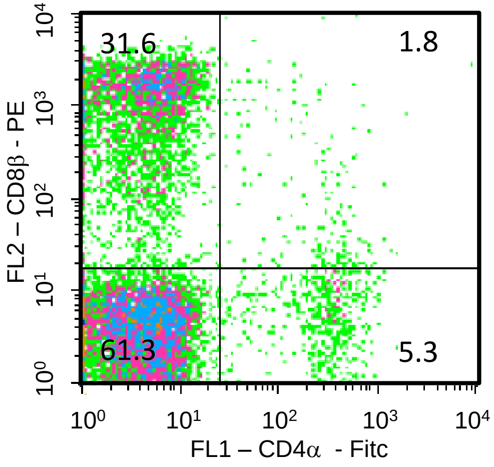

Plot 18: Fluorescence density dot-plot (gate L)

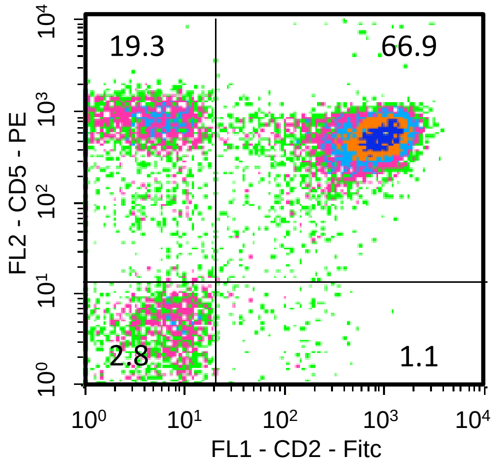

Jejunal intraepithelial cells (piglet aged 25 days, no feeding group)

Plot 19: Density dot-plot (morphological)

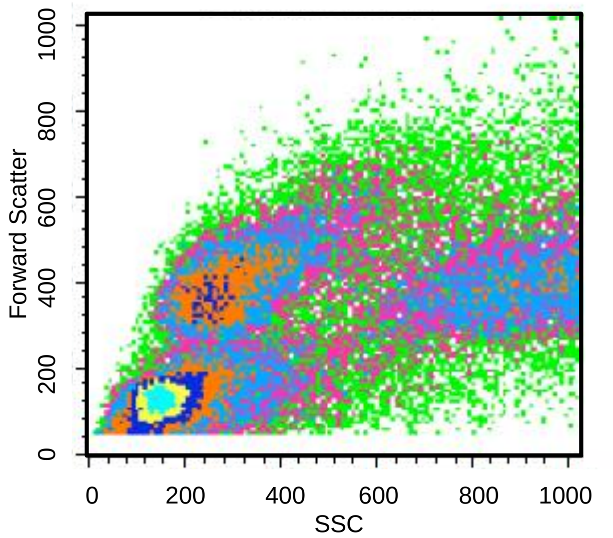

Plot 20: Fluoresecence density dot-plot of unstained cells (negative control)

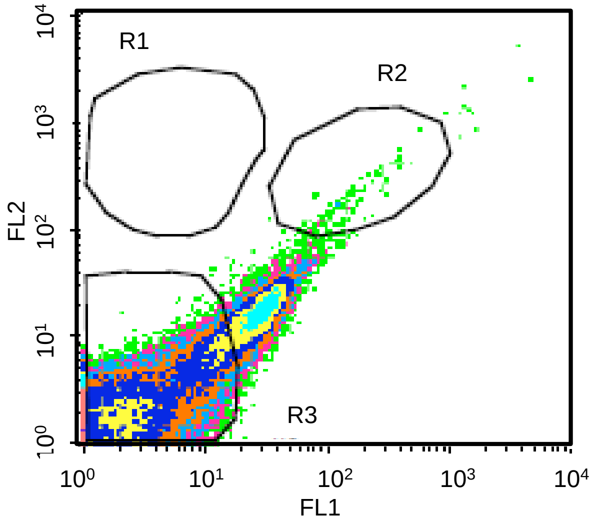

Jejunal intraepithelial cells (identical sample as in plots 19-20)

Plot 21: Fluorescence density dot-plot

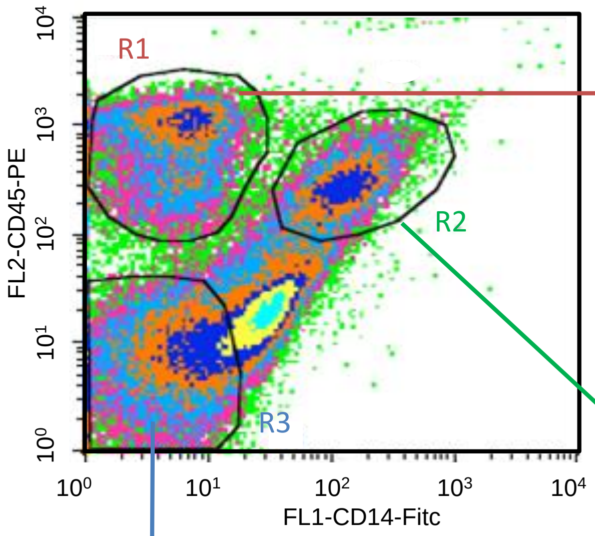

Plot 22: Density dot plot (R1 morphological)

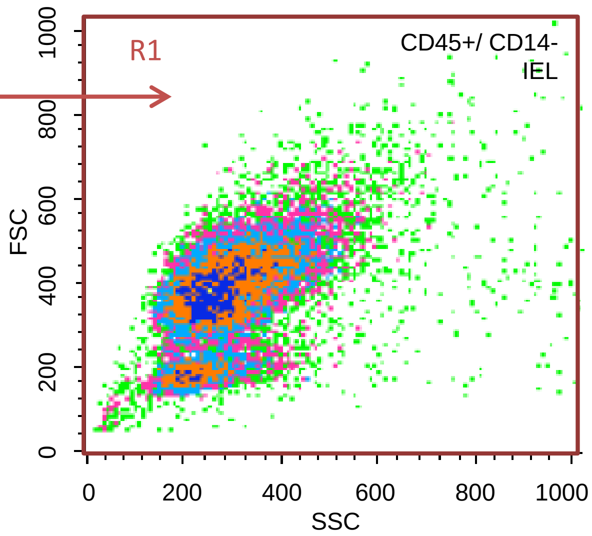

Plot 23: Density dot-plot (R3, morphological)

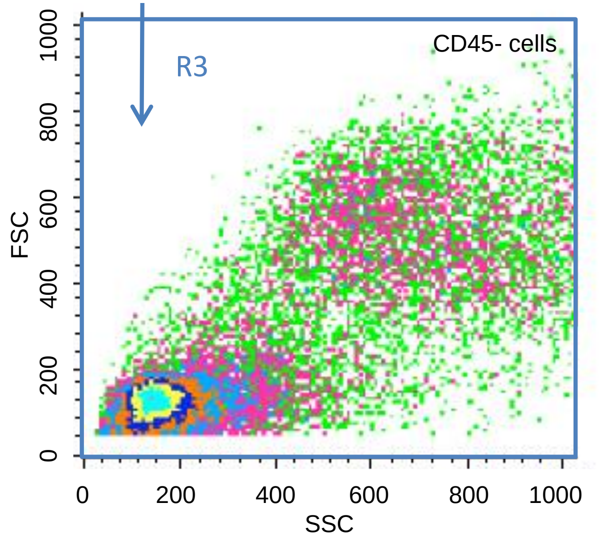

Plot 24: Density dot-plot (R2 morphological)

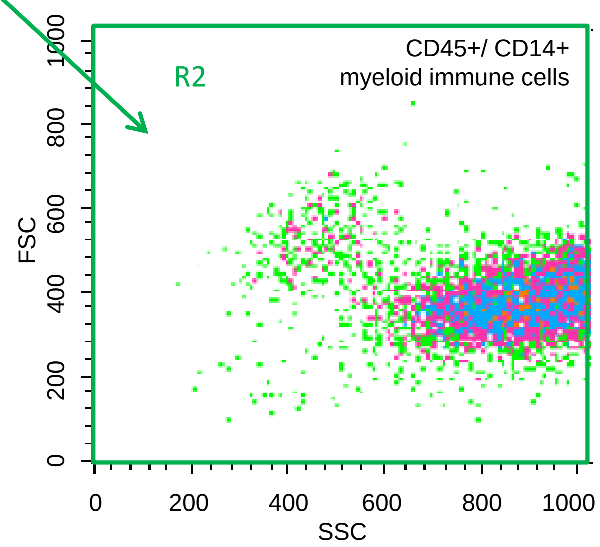

Ileal MLN

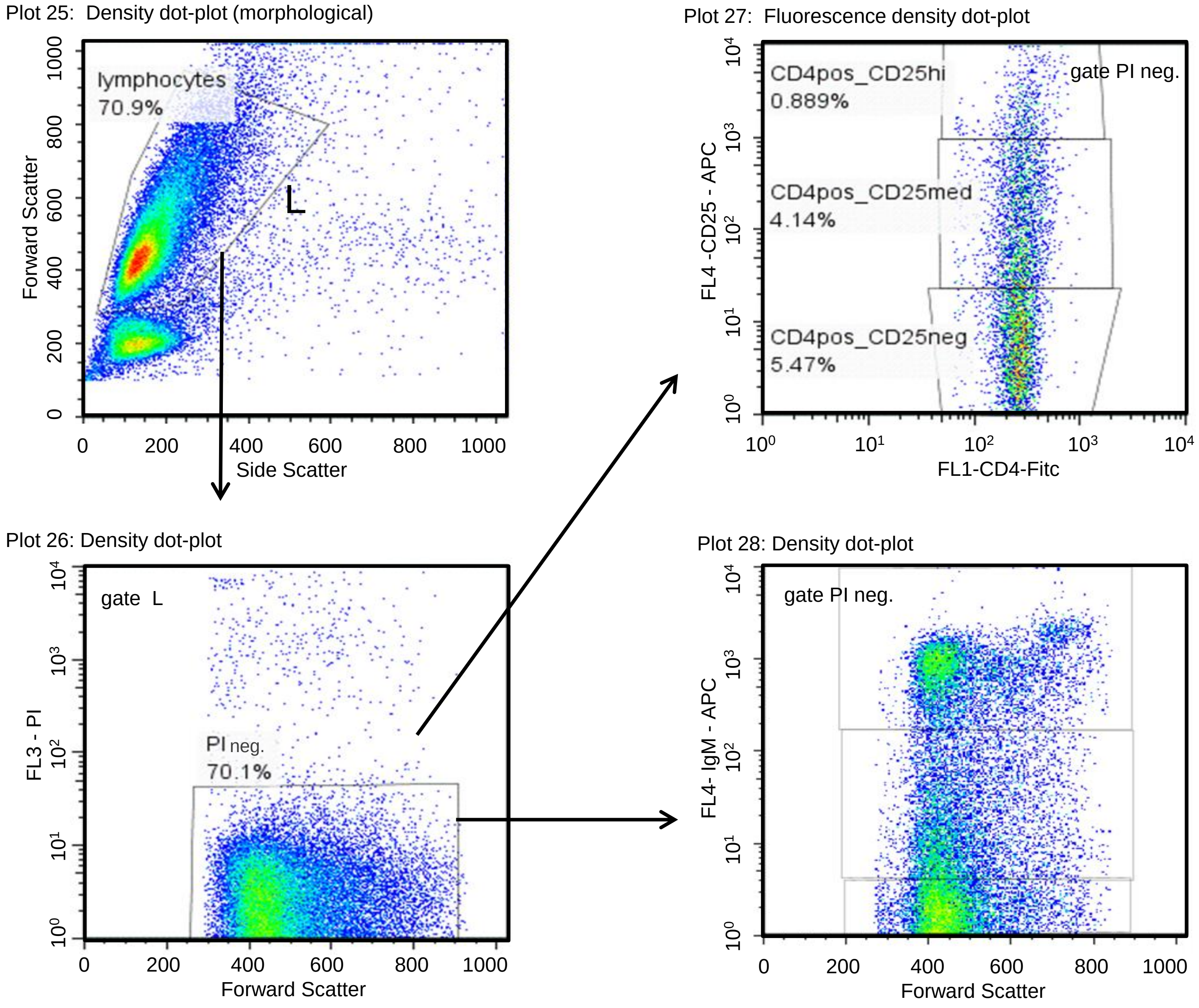
Plot 25: Density dot-plot (morphological)
lymphocytes
70.9%
L
Forward Scatter
Side Scatter
0
200
400
600
800
1000
Plot 27: Fluorescence density dot-plot
CD4pos_CD25hi
0.889%
gate PI neg.
CD4pos_CD25med
4.14%
CD4pos_CD25neg
5.47%
FL4 -CD25 - APC
FL1-CD4-Fitc
10^0
10^1
10^2
10^3
10^4
Plot 26: Density dot-plot
gate L
PI neg.
70.1%
FL3 - PI
Forward Scatter
Plot 28: Density dot-plot
gate PI neg.
FL4- IgM - APC
Forward Scatter
