# Supplementary material for: Probiotic Treatment Decreases the Number of CD14-Expressing Cells in Porcine Milk Which Correlates with Several Intestinal Immune Parameters in the Piglets
Source: Front Immunol. 2015 Mar 10;6:108. doi: 10.3389/fimmu.2015.00108 (PMC4354412; doi:10.3389/fimmu.2015.00108)
Supplement: Supplementary file 1 [file data_sheet_1.zip › Table_S1.docx]

Supplementary data, Table 1:

**Portion of CD14^+^ /MHCII^-^ cells (% of all cells) in the milk of sows**

**at different days of lactation**

|  |  |  |  |  |  |
| --- | --- | --- | --- | --- | --- |
|  |  |  |  |  |  |

|  | day 7 | | day 17 | | day 28 | |
| --- | --- | --- | --- | --- | --- | --- |
|  | Control | *E. faecium* | Control | *E. faecium* | Control | *E. faecium* |
|  | 14,7 | 17,9 | 50,5 | 22,0 | 48,0 | 4,6 |
|  | 12,1 | 22,9 | 25,8 | 20,7 | 21,4 | 25,8 |
|  | 48,4 | 6,0 | 32,4 | 6,2 | 20,4 | 16,7 |
|  | 15,0 | 26,9 | 19,8 | 23,1 | 15,6 | 32,6 |
|  | 34,4 | 21,3 | 46,2 | 6,4 | 64,1 | 20,1 |
|  | 36,4 |  | 26,7 | 18,8 | 57,5 | 58,8 |
|  | 20,6 |  | 28,9 | 11,4 | 55,6 | 60,7 |
|  |  |  | 10,2 | 10,6 | 57,5 | 6,1 |
|  |  |  | 19,5 | 15,8 | 73,2 | 15,3 |
|  |  |  | 22,9 | 6,9 | 22,3 | 33,5 |
|  |  |  | 29,6 | 21,3 | 19,3 | 14,4 |
|  |  |  | 34,6 | 10,1 | 34,1 | 29,3 |
|  |  |  |  |  |  |  |
| AM | 25,9 | 19,0 | 28,9 | 14,4 | 40,7 | 26,5 |
| SD | 13,9 | 8,0 | 11,2 | 6,5 | 20,7 | 18,1 |

Notes:

AM, Arithmetic Mean

SD, Standard Deviation
